# Chronic Kratom Use as a Potential Risk Factor for Embolic Stroke of Undetermined Source: A Case Report

**DOI:** 10.7759/cureus.111385

**Published:** 2026-06-23

**Authors:** Zhuo Luan, Seunghong Rhee, Paisith Piriyawat, Amy H Sim

**Affiliations:** 1 Neurology, Paul L. Foster School of Medicine, Texas Tech University Health Sciences Center El Paso, El Paso, USA; 2 Radiology, Paul L. Foster School of Medicine, Texas Tech University Health Sciences Center El Paso, El Paso, USA

**Keywords:** a risk factor, embolic stroke of undetermined source, etiologies of stroke, ischemic stroke, kratom

## Abstract

Kratom (*Mitragyna speciosa*) is an herbal supplement that has gained popularity for its opioid-like and stimulant effects. Despite its widespread use, the U.S. Food and Drug Administration has issued warnings about its potential toxicity and the risk of cardiovascular and neurological complications. While a range of neurological effects has been reported, its link to ischemic stroke remains unclear. We report a 39-year-old man who developed an embolic stroke of undetermined source (ESUS) after using kratom daily for two years, with no other identifiable risk factors. The patient demonstrated transient ischemia of the middle cerebral artery that resolved spontaneously, associated with multifocal ischemic infarcts on MRI. Extensive cardiac, vascular, and hypercoagulable testing failed to identify an alternative cause. To our knowledge, this is the first reported case of ESUS in the context of chronic kratom use. This case highlights a potential association between long-term kratom consumption and ischemic stroke and underscores the need for further studies to better understand its cerebrovascular effects and possible mechanisms.

## Introduction

Embolic stroke of undetermined source (ESUS) is a clinically important subtype of cryptogenic ischemic stroke, defined by non-lacunar infarction in the absence of a major-risk cardioembolic source, significant proximal arterial stenosis, or other clearly identifiable etiologies after a comprehensive diagnostic evaluation [1]. ESUS accounts for approximately 17% of ischemic strokes and is particularly prevalent among younger individuals, in whom traditional vascular risk factors may be absent [2]. The underlying mechanisms of ESUS are heterogeneous and may include occult cardioembolism, nonstenotic atherosclerotic plaque, patent foramen ovale, cancer, and hypercoagulability [2]. Identifying novel or underrecognized risk factors is therefore critical for improving diagnostic precision and guiding secondary prevention.

Kratom, a substance derived from the tropical tree *Mitragyna speciosa*, has become increasingly popular in recent years, especially in the United States. Many people use it to manage chronic pain, improve mood, or cope with opioid withdrawal [3]. Despite its growing use, kratom is largely unregulated, and scientists are still uncovering its potential risks [3]. The U.S. Food and Drug Administration has issued warnings about possible toxicity and other adverse health effects, highlighting the need for caution [4].

Kratom has been linked to a range of neurological effects, including seizures, encephalopathy, and reversible changes in white matter [5]. Some case reports describe temporary cerebrovascular issues, such as conditions resembling posterior reversible encephalopathy syndrome (PRES), suggesting that kratom may affect blood vessel tone and endothelial function [6]. There is also emerging evidence that kratom can impact the heart and autonomic nervous system, potentially causing arrhythmias or unstable blood pressure, which could further influence cerebrovascular risk [7]. Despite these findings, direct evidence connecting kratom use to ischemic stroke is very limited, and to date, no cases have specifically reported a link to ESUS.

We present a case of ESUS in a young patient with chronic kratom use and no identifiable etiology after extensive evaluation. To our knowledge, this represents the first documented case of ESUS potentially associated with long-term kratom exposure.

## Case presentation

A 39-year-old Hispanic man with no significant past medical history presented with an acute-onset headache followed by left-sided numbness and weakness involving the arm and leg approximately two hours prior to arrival at an outside hospital. By the time he arrived, his symptoms had partially improved, with only residual numbness in the left arm and leg. Given the mild and improving deficits, with a National Institutes of Health Stroke Scale (NIHSS) score of 1, he was not considered a candidate for thrombolysis with tenecteplase. An initial non-contrast CT of the head showed no acute intracranial abnormalities. CT angiography (CTA) of the head and neck reportedly demonstrated a non-occlusive thrombus in the right middle cerebral artery; however, the original images were not available for review.

Upon transfer to our institution, the patient was awake, alert, and fully oriented, with fluent speech and complete resolution of prior focal neurological deficits, including the left-sided numbness. A repeat non-contrast CT head (Figure 1A) showed no acute intracranial abnormalities. Repeat CTA (Figure 1B) demonstrated complete resolution of the previously reported thrombus, and CT perfusion imaging showed no perfusion deficits (Figure 2A and Figure 2B).

**Figure 1:**
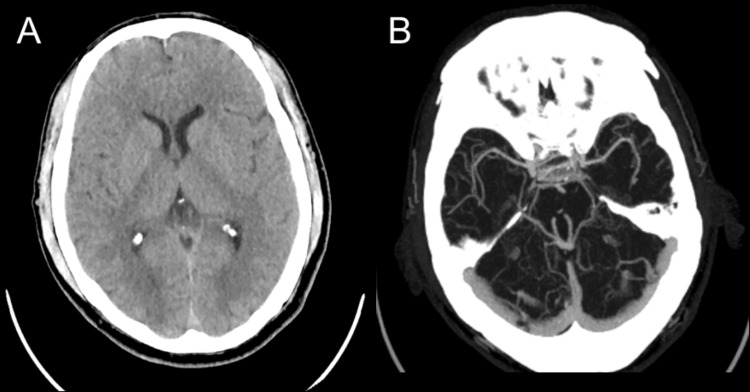
Initial neuroimaging on admission Non-contrast CT (A) demonstrates no acute intracranial hemorrhage. CTA of the head (B) demonstrates no evidence of large-vessel occlusion. CTA, CT angiography

**Figure 2:**
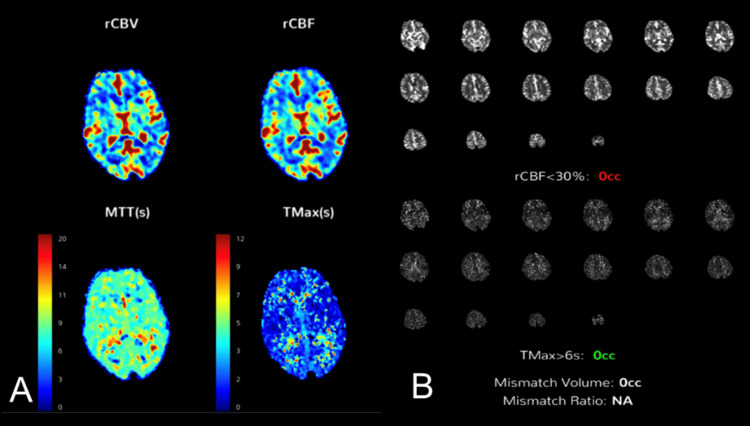
Initial neuroimaging at admission CT perfusion maps (A), including CBV, CBF, MTT, and TTP, demonstrate symmetric and normal perfusion. CT perfusion-derived rCBF and Tmax images (B) confirm the absence of perfusion deficits. CBV, cerebral blood volume; CBF, cerebral blood flow; MTT, mean transit time; TTP, time-to-peak; rCBF, relative cerebral blood flow

MRI of the brain with and without contrast, obtained on hospital day 2, revealed multiple acute ischemic infarcts involving the right frontal, parietal, and occipital lobes, predominantly in watershed distributions. Additional punctate infarcts were identified in the left frontal and parietal lobes, as well as the right temporal lobe, consistent with a multifocal embolic process (Figure 3).

**Figure 3:**
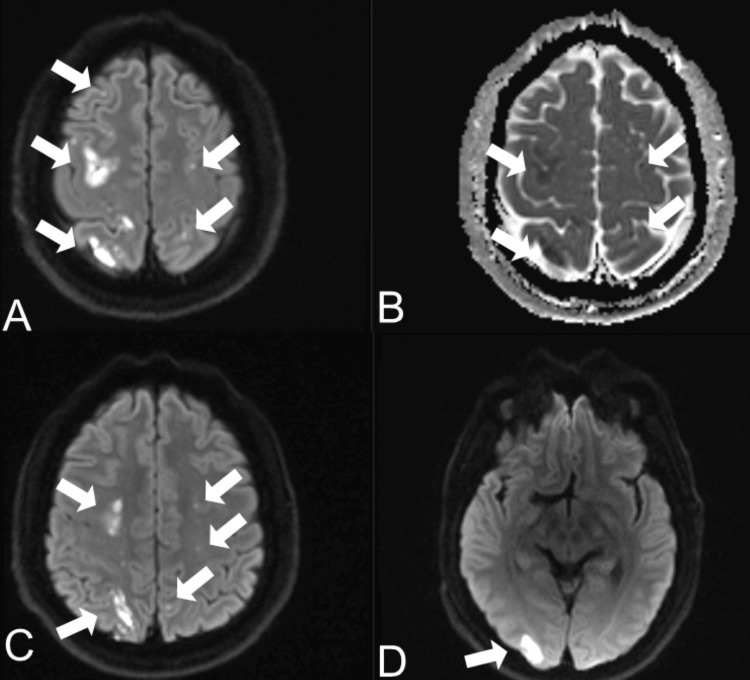
Brain MRI on hospital day 2 demonstrating multiterritorial ischemic infarcts Axial DWI (A) reveals focal diffusion restriction in the bilateral frontal and parietal lobes, with right-sided predominance (arrows), confirmed by true restricted diffusion on the corresponding ADC map (B). Additional DWI images demonstrate infarcts in the bilateral frontal and parietal lobes (C) and the right occipital lobe (D). DWI, diffusion-weighted imaging; ADC, apparent diffusion coefficient

An extensive diagnostic evaluation was performed. Electrocardiography and continuous telemetry demonstrated a normal sinus rhythm without evidence of arrhythmia. Transthoracic echocardiography with bubble study showed normal cardiac structure and function, with an ejection fraction of 60%-65%. Transesophageal echocardiography revealed no left atrial appendage thrombus or interatrial shunt.

Vascular imaging, including CTA and carotid duplex ultrasonography, showed no significant stenosis or dissection. However, nonstenotic atherosclerotic plaques were identified, including a soft plaque in the right internal carotid artery (Figure 4A and Figure 4C) and a calcified plaque in the common carotid artery (Figure 4B and Figure 4D). The soft plaque had a smooth surface and lacked high-risk features, making it less likely to be the primary stroke source, particularly given the bilateral involvement of anterior and posterior vascular territories.

**Figure 4:**
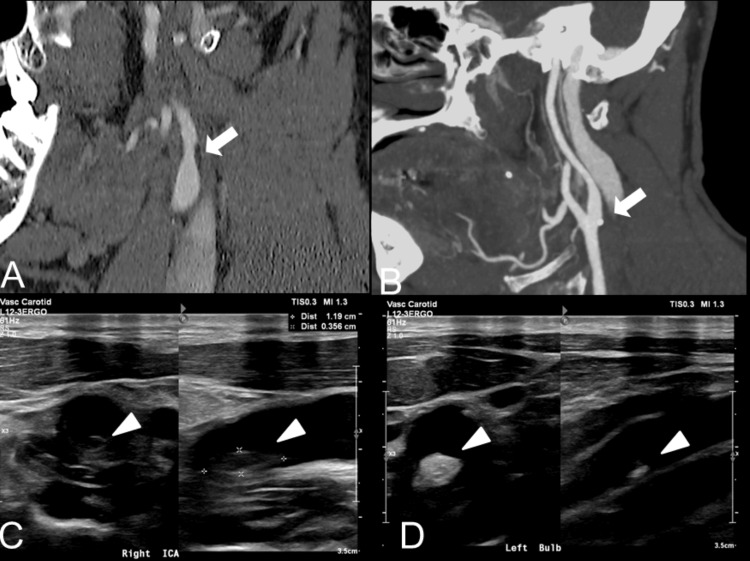
CTA and carotid duplex ultrasonography demonstrating bilateral nonocclusive atherosclerotic disease of the carotid arteries CTA of the right internal carotid artery (A) shows a smooth-surfaced, lipid-rich plaque (arrow) with a Hounsfield unit of approximately 42, corresponding to a hypoechoic plaque (arrowhead) on carotid ultrasound (C), measuring 1.19×0.36 cm. CTA of the left carotid bulb (B) demonstrates a small calcified plaque (arrow), corresponding to a hyperechoic plaque (arrowhead) on carotid ultrasound (D). CTA, CT angiography

A comprehensive hypercoagulable workup, including antiphospholipid antibodies, Factor V Leiden mutation, prothrombin gene mutation, protein C, protein S, and antithrombin III levels, was unremarkable (Table 1). Laboratory studies were notable only for elevated triglycerides (380 mg/dL) (Table 1). Urine toxicology screening was negative. Further history revealed daily consumption of kratom extract beverages for approximately two years. The patient denied tobacco, alcohol, or illicit drug use.

**Table 1:** Laboratory results Laboratory testing revealed hypertriglyceridemia, while the comprehensive hypercoagulable workup was negative. HDL, high-density lipoprotein; LDL, low-density lipoprotein; IgA, immunoglobulin A; IgG, immunoglobulin G; IgM, immunoglobulin M; APL-U/mL, IgA phospholipid units per milliliter; GPL-U/mL, IgG phospholipid units per milliliter; MPL-U/mL, IgM phospholipid units per milliliter

Blood/serum	Value	Reference
Prothrombin time	14.8 s	11.8-14.8 s
International normalized ratio	1.2	0.9-1.1
Cholesterol	196 mg/dL	0-199 mg/dL
Triglyceride	380 mg/dL	0-150 mg/dL
HDL cholesterol	46 mg/dL	40-60 mg/dL
Total/HDL ratio	4.3	1-5
LDL	74 mg/dL	50-100 mg/dL
A1c	5.3%	<5.7%
Factor V Leiden (R506Q)	Not detected	Not detected
Prothrombin (factor II) (G20210A variant)	Not detected	Not detected
Antithrombin III activity	92%	80-135%, normal
Antithrombin III antigen	83%	80-120%, normal
Cardiolipin Ab (IgA)	6.4 APL-U/mL	<20.0 APL-U/mL
Cardiolipin Ab (IgG)	<2.0 GPL-U/mL	<20.0 GPL-U/mL
Cardiolipin Ab (IgM)	3.6 MPL-U/mL	<20.0 MPL-U/mL
Protein C, antigen	98%	70-140%, normal
Protein S antigen, total	84%	70-140%, normal
Protein S antigen, free	112	57-171%, normal
Protein S, activity	113	70-150%, normal

Based on the clinical presentation, imaging findings, and unrevealing diagnostic evaluation, the patient was diagnosed with ESUS. He was started on aspirin and statin therapy and was counseled to discontinue kratom use.

## Discussion

This case describes a young patient with multiple ischemic infarcts affecting different brain regions, consistent with ESUS in the setting of chronic kratom use. The involvement of multiple vascular territories strongly supports an embolic mechanism. Although bilateral non-stenotic carotid plaques were present and are generally considered low risk, and the patient also had hypertriglyceridemia, a comprehensive evaluation failed to identify a clear source, including major cardioembolic causes, significant arterial stenosis, or a hypercoagulable state, thereby supporting the diagnosis of ESUS as defined by prior criteria [8].

This is, to our knowledge, the first reported case of ESUS potentially associated with chronic kratom use. While a causal relationship cannot be established from a single case, the temporal association and absence of an alternative explanation raise important questions about its potential cerebrovascular effects. Although the literature on kratom increasingly recognizes neurological complications such as seizures and PRES [5], there is very limited evidence directly linking it to ischemic stroke.

Several biologically plausible mechanisms could explain the potential association between kratom use and vascular or cerebrovascular effects. The main alkaloids in kratom, such as mitragynine, interact with multiple receptor systems, including opioid, adrenergic, and serotonergic pathways [9]. These interactions may influence autonomic signaling and vascular responses, potentially leading to vasoconstriction and endothelial dysfunction [10]. Adrenergic stimulation, in particular, promotes vasoconstriction [11], while serotonergic pathways play a role in regulating vascular tone and maintaining endothelial integrity [12]. Together, these effects could help explain reports of multi-organ toxicity and cardiotoxicity associated with kratom, including coronary atherosclerosis, cardiac arrhythmias, myocardial infarction, hypertensive cardiovascular disease, and cardiopulmonary arrest [7]. Although direct clinical evidence is limited, these mechanisms raise the possibility that kratom use could contribute to cerebrovascular events, potentially through increased embolic risk or cerebral artery atherosclerosis.

Kratom may exert cardiovascular effects that could contribute to embolic risk. Prior studies have reported cardiac arrhythmias, hypertension, and endothelial dysfunction associated with kratom exposure [7]. Although no arrhythmia was detected during hospitalization in this case, paroxysmal atrial fibrillation remains a well-recognized occult cause of ESUS and cannot be excluded without prolonged cardiac monitoring.

Another important consideration is the presence of non-stenotic carotid atherosclerotic plaques. Emerging evidence suggests that even plaques causing <50% luminal narrowing, particularly those with high-risk features such as lipid-rich necrotic cores, can act as potential embolic sources [13]. In this patient, both soft and calcified plaques were present, including a lipid-rich component, but the smooth surface and absence of other high-risk features suggest a relatively low overall risk of instability. Hypertriglyceridemia represents an additional vascular risk factor that may contribute to atherosclerosis and endothelial dysfunction. Elevated triglyceride levels are associated with atherogenic remnant lipoproteins and increased vascular inflammation, which can accelerate plaque formation and destabilization [14]. While atherosclerosis generally develops over years, chronic exposure to vasoactive substances such as kratom could plausibly exacerbate endothelial injury on top of pre-existing hypertriglyceridemia, similar to its proposed role in coronary atherosclerosis. Taken together, the combination of carotid plaque, hypertriglyceridemia, and the potential vascular effects of kratom may create a vascular environment more susceptible to cerebrovascular events.

Despite these possible mechanisms, clinical evidence directly linking kratom use to ischemic stroke remains extremely limited. Nevertheless, this case adds to the emerging literature on the systemic effects of kratom and highlights its potential role in cerebrovascular disease. It underscores the importance of heightened clinical awareness, particularly in younger patients presenting with cryptogenic stroke and a history of kratom use. Future studies, including pharmacovigilance analyses, prospective cohort studies, and mechanistic investigations, are needed to better characterize the vascular risks associated with kratom and to determine whether a causal relationship exists.

## Conclusions

This case highlights the possibility that chronic kratom use may represent a potential, though unproven, risk factor for ESUS. To our knowledge, this is the first reported case linking long-term kratom use to ESUS. Clinicians should consider kratom exposure in the evaluation of patients presenting with unexplained stroke, particularly in younger individuals without traditional vascular risk factors. Further studies are needed to determine whether a causal relationship exists.
